# Synaptic deficits in sepsis: role of glial cells

**DOI:** 10.1186/cc12989

**Published:** 2013-11-05

**Authors:** Carolina A Moraes, Gabriel dos Santos, Joana D'Avila, Pedro Perdigão, Tânia Spohr, Flávia Lima, Newton Castro, Claudia Benjamim, Fernando Augusto Bozza, Flávia Carvalho Alcantara Gomes

**Affiliations:** 1 Institute Oswaldo Cruz, FIOCRUZ, Rio de Janeiro, Brazil; 2 Institute of Biomedical Sciences, Federal University of Rio de Janeiro, Brazil

## Background

Recent clinical studies have shown that sepsis survivors can develop long-term cognitive impairment. The cellular and molecular mechanisms involved in these events are not yet completely understood. In this study, we investigate the synaptic deficits in sepsis and the involvement of glial cells in this process.

## Materials and methods

Using a clinically relevant model of sepsis (cecal ligation and puncture), we observed a decrease of recognition memory 9 days after sepsis. At the same time, by colocalization between pre-synaptic and post-synaptic protein, synaptophysin and PSD-95, we observed a reduction of structural synapses in the hippocampus and cerebral cortex of septic mice. To define the molecular mechanisms accountable for synaptic loss in sepsis, we used an *in vitro *approach treating neuronal cultures with conditioned medium from astrocyte (ACM) and microglial (MCM) cultures stimulated with LPS.

## Results

We observed that the MCM reduced the synapse number and the ACM increased the number of synapses. The analysis of conditioned medium composition showed that MCM had increased levels of IL-1β while the ACM had increased levels of TGF-β1, as compared with medium from the non-LPS-stimulated cultures. The increased levels of IL-1β, from microglial activated with LPS, accompanied by an increase of TGF-β1, from LPS-activated astrocytes, suggests an anti-synaptic activity in IL-1β and pro-synaptic actions in TGF-β1. Inhibition assays with the addition of soluble IL-1β receptor (IL-1 Ra) prevented the MCM-induced synapsis loss. To understand whether the loss in synapse density would have functional outcomes we performed patch clamp experiments in neurons treated with microglia conditioned medium (MCM) and MCM of LPS-stimulated cultures. Patch-clamp recordings in the MCM-treated neurons showed a reduction in postsynaptic current frequency, while an increase in current amplitudes suggests a functional synaptic deficit.

## Conclusions

These findings show, for the first time, a correlation between synaptic deficits and memory dysfunction, suggesting a possible mechanism for cognitive impairment after sepsis as well as a glial-derived molecule mediating synapse reduction.

